# Waiting times for outpatient visits during military conflict: An observational study

**DOI:** 10.1371/journal.pone.0313301

**Published:** 2025-04-16

**Authors:** Jacob Dreiher, Sharon Einav, Shlomi Codish, Amit Frenkel

**Affiliations:** 1 Hospital Administration, Soroka University Medical Center, and The Faculty of Health Sciences, Ben-Gurion University of the Negev, Beer-Sheva, Israel; 2 General Intensive Care Unit, Shaare Zedek Medical Center, and Anesthesia and Intensive Care Medicine, Hebrew University-Hadassah Faculty of Medicine, Jerusalem, Israel; 3 General Intensive Care Unit, Soroka University Medical Center, and The Faculty of Health Sciences, Ben-Gurion University of the Negev, Beer-Sheva, Israel; Ladoke Akintola University of Technology Teaching Hospital: LAUTECH Teaching Hospital, NIGERIA

## Abstract

**Background:**

Environmental events, including military conflicts, may dramatically affect a hospital’s ability to provide routine treatments while maintaining reasonable waiting times.

**Objective:**

To examine the impact of a military conflict (“Protective Edge”, PE) on the volume of activity and waiting times for outpatient clinics in a tertiary medical center.

**Methods:**

Outpatient visits during PE (July-August 2014) were compared to outpatient visits during July-August 2013 (pre-conflict period) and 2015 (post-conflict period) with regards to the daily number of visits and waiting times. Clinics with at least 5,000 annual visits were included. Quantile regression adjusted for confounders was used for the multivariable models, in a stratified analysis by specialty.

**Results:**

There were 87,495 outpatient visits during PE and 197,029 visits during the pre- and post-conflict periods. An 11% decrease in the daily number of visits was noted (ranging from 6% decrease in oncology and cardiology to 19–20% decrease in psychiatry and pediatrics). During PE, statistically significant longer waiting times were found for surgery (+1.0 day) and imaging (+1.1 days), while a 2.4 days decrease was noted in pediatrics, controlled for age, sex, ethnicity and the daily number of visits. Median waiting times were unchanged for cardiology, medicine, psychiatry and cardiology.

**Conclusions:**

In the midst of a continuing military conflict, there was a notable increase in outpatient visit waiting times in some disciplines, but not all, despite a reduction in the overall volume of visits. Investigating whether similar impacts on patient care occur during other military conflicts or pandemics necessitates further research.

## Introduction

Complex emergencies affect health-care delivery in four main areas; the workforce, infrastructure, information access, and organization of health services [1]. When the strain of a public healthcare crisis is added to an already overloaded healthcare system, the quality of healthcare service delivered to fragile special populations is often very much affected [2]. The medical literature is rife with descriptions of the effects of public healthcare crises on vulnerable populations. A systematic review of data from 181 countries (2000–2019) estimated excess deaths due to conflicts and showed major increases in maternal and infant mortality and decreases in critical vaccinations (DPT, measles) up to 7 years post-conflict [3]. Most studies published so far deal with either emergency preparedness in terms of acute care [4–9] or the actual impact of a military conflict on acute care [10–13].

Outpatient clinics usually deal with non-urgent medical care and are highly dependent on efficient organization. As such their waiting times are very likely to be affected during such crises.

Waiting time is an important component of patient satisfaction with the service provided at outpatient clinics [14,15]. However, more importantly, reduced outpatient waiting times have been associated with better doctor-patient communication [16] and health outcomes [17].

Achieving reasonable waiting times remains a delicate balancing act which can easily be disrupted by unplanned public health crises. Treating clinicians may be less available for some services and patients may be unable or reluctant to attend others [18,19].

Operation Protective Edge was a military conflict between Israel and Hamas, taking place during the summer of 2014, from July to August. The operation was launched by the Israeli Defense Forces in response to escalating rocket fire from Gaza. It involved significant aerial, naval, and ground operations aimed at neutralizing Hamas’ military infrastructure and rocket-launching capabilities. The conflict resulted in widespread casualties and displacement, particularly in Gaza, and led to increased security threats across southern and central Israel, where civilians faced regular air raid sirens and rocket attacks.

The aim of the current study was to examine waiting times for outpatient visits of patients during a military conflict vs. during comparable times in the years before and after. We hypothesized that waiting times for more elective care (e.g., dermatology) will be prolonged during military conflicts, while waiting times for more protocolized care with strict timing for treatment (e.g., oncology) would not be affected, and could even shorten.

## Materials and methods

Following Institutional Review Board of Soroka University Medical Center (SUMC) approval (protocol number 0173-19-SOR) we conducted a retrospective analysis of data collected in real-time at SUMC.

SUMC, located in the city of Beer-Sheva, 40 kilometers from the Gaza strip, is a major referral center and the only tertiary hospital in southern Israel. It serves an estimated population of 1,000,000 people. We selected Protective Edge (PE) as a case study, since during this period (July-August 2014) more than 320 rockets were launched at the Be’er Sheva region. The hospital served as the frontline and referral center for those injured while continuing to cover the regular needs of its catchment population.

The hospital currently has 1,173 admission beds and during the study period, the annual number of visits to the emergency department was about 100,000. SUMC is the largest hospital owned by Clalit Healthcare, the sick fund covering about 52% of the population of Israel.

For the purpose of the current study, we included all visits to the outpatient clinics of SUMC during the military conflict known as PE (July-August 2014) and compared them to all visits to outpatient clinics in the equivalent months in pre-conflict period (July-August 2013) and the post-conflict period (July-August 2015), jointly defined as the comparison period.

Relevant data were extracted from the files of all the patients that had been registered for treatment as outpatients in SUMC during the study period. Repeat visits were treated as separate cases.

The primary outcome measure was waiting time for the outpatient visits, defined as the number of days since the request for the visit was received to the actual day of the visit. Waiting times during the military conflict were compared to waiting times during the comparison period. Secondary outcome measures included a comparison of the characteristics of the patients and number of visits. We collected data on patient demographics (age, sex), waiting times and type of clinic.

All data presented in the current study were extracted from the database of SUMC, which includes data going backwards about 20 years. Clalit Healthcare uses several functional databases (administrative, medical and logistic). All Clalit data are imputed in real time by the administrative, medical and logistical staff. The data are all stored on a central Structured Query Language (SQL)-based platform and SAP Business Objects software is used to pull the full data for reports.

We assumed that during the study period there would be an increase in waiting time of 10% over previous years. Waiting time was estimated as 30±40 days based on prior data. We included clinics with more than 5,000 annual visits in 2013 in the analysis of specific clinic types. Assuming a significance level of 5%, our study would have a power of 95% to detect a 10% difference in waiting time.

Cases with missing data constituted approximately 0.4% of the visits. These included visits with ID numbers that could not be validated against the national population registry and lacked data on demographics (e.g., age).

We grouped similar categories of visits into seven main categories of clinics. For example, general surgery and orthopedic surgery were grouped as “surgery”, while endocrinology and rheumatology were grouped as “medicine”.

All data analyses were performed using SPSS Statistical Software (IBM Corporation) version 29. Descriptive statistics were carried out using frequencies for categorical data. As continuous variables were not normally distributed (according to Kolmogorov-Smirnov test) we report medians and interquartile ranges to describe them. Univariate analyses were performed using nonparametric tests (Kruskal-Wallis test) for continuous data. The Chi-square test was used for the associations between categorical variables. Significance of statistical results in a two-sided test was defined as <5%. Multivariable analyses for waiting times were performed using quantile regression (Enter method). We examined the waiting time during PE compared to 2013, and also compared waiting times in 2015–2013, controlled for confounders, to reflect secular trends. Several quantiles were analyzed, to reflect the differential effect of PE in different quantiles. Parameter estimates for waiting times in 2014 and 2015 vs. 2013, adjusted for covariates were plotted as quantiles with their 95% confidence intervals, for each clinic type. The data were extracted from an administrative database with a limited number of variables; therefore, all variables were included in the model. Interactions between clinic type and period were included in the models as well.

## Results

**Fig 1:**
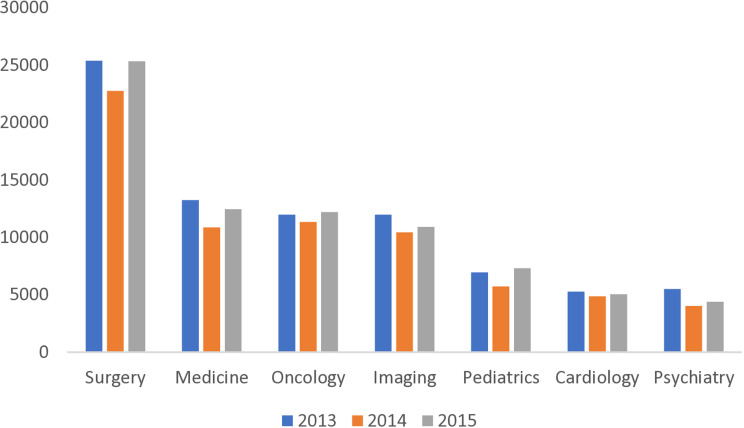
Number of visits per clinic type, 2013-2015.

In total, 87,495 outpatient visits took place between July 1, 2014 and August 31, 2014. In comparison, 197,029 visits took place during the pre- and post-conflict periods. This translates into a daily average of 1,996 visits during Protective Edge military operation, vs. 2,246 in the pre-conflict and 2,236 in the post-conflict periods, i.e., an 11% decrease. There was significant variability between clinics, from 6% decrease in oncology and cardiology to 19% decrease in psychiatry and 20% decrease in pediatrics (Fig 1).

*Primary outcome measure* – The unadjusted median waiting time 19 days during PE, vs. 20 days in the pre-conflict period and 18 days in the post-conflict period (p<0.001) (Table 1).

**Table 1:** Patient characteristics for outpatient visits, 2013-2015.

Characteristic	2013 Pre-conflict period N=98,873	2014 Protective Edge military operation N=87,495	2015 Post-conflict period N=98,156	P for trend
Male sex	44,003 (44.5%)	39,389 (45.0%)	42,879 (43.7%)	<0.001
Age (median, IQR)	52 (27–66)	51 (27–66)	50 (26–66)	<0.001
Waiting time (median, IQR) (n=223,197)	20 (7–40)	19 (7–42)	18 (7–42)	0.312
Number of visits per day (median, IQR)	2,283 (2,108–2,419)	2,017 (1,912–2,164)	2,243 (2,044–2,391)	<0.001

There was marked variability between clinics, with median waiting times ranging from 13 days for psychiatry to 38 days for medicine during PE (Table 2).

**Table 2:** Median waiting time for outpatient clinics, by clinic type, n=223,179), Univariate quantile regression.

Type of Clinic	2013 N=98,873	2014 N=87,495		2015 N=98,156	
	Median waiting time (days)	Median waiting time (days)	Difference (95% CI)	Median waiting time (days)	Difference (95% CI)
Surgery	17	18	**1 (0.5; 1.5)**	15	**-2 (-2.5; -1.5)**
Medicine	38	35	**-3 (-4.7; -1.3)**	40	**2 (0.3; 3.7)**
Oncology	20	19	**-1 (-1.7; -0.3)**	19	0 (-0.7; 0.7)
Imaging	18	17	**-1 (-1.7; -0.3)**	13	**-5 (-5.7; -4.3)**
Pediatrics	22	21	-1 (-2.7; 0.7)	21	-1 (-2.6; 0.6)
Cardiology	25	23	**-2 (-3.4; -0.6)**	25	0 (-1.4; 1.4)
Psychiatry	11	13	**2 (1.0; 3.0)**	11	0 (-0.9; 0.9)

Adjusted median waiting time was significantly longer during the military operation (Table 3) for certain clinics (surgery: +1.0 day, 95% CI: 0.5–1.6; imaging: +1.1 days, 95% CI: 0.4–1.9) and shorter by 2.4 days for pediatrics (95% CI: -4 to -0.8 days), adjusted for age, sex, ethnicity and the number of daily visits. No significant changes were noted for medicine, oncology, cardiology and psychiatry (Table 3).

**Table 3:** Median waiting time for outpatient clinics (Multivariable quantile regression model, n=223,179).

Type of Clinic	Change in median waiting time, Protective Edge vs. pre-conflict (95% CI)	Change in median waiting time, Post-conflict vs. pre-conflict (95% CI)	P for interaction between period and clinic time
Surgery	**1.0 (0.5; 1.6)**	**−1.3 (-1.9; −0.8)**	**<0.001**
Medicine	−0.3 (-2.1; 1.6)	**3.9 (2.1; 5.6)**	**<0.001**
Oncology	0.4 (-0.3; 1.1)	0.1 (–0.6;1.1)	0.372
Imaging	**1.1 (0.4;1.9)**	**−4.1 (**–**4.8;-3.4)**	**<0.001**
Pediatrics	**−2.4 (-4.0;-0.8)**	**−1.8 (**–**3.1; −0.4)**	**0.039**
Cardiology	0 (–1.3; 1.3)	**1.6 (0.3;2.9)**	**<0.001**
Psychiatry	0 (–1.1;1.1)	−0.1 (–1.1;0.8)	0.135

Parameter estimates for the years are controlled for age, sex, ethnicity and the daily number of visits

**Fig 2:**
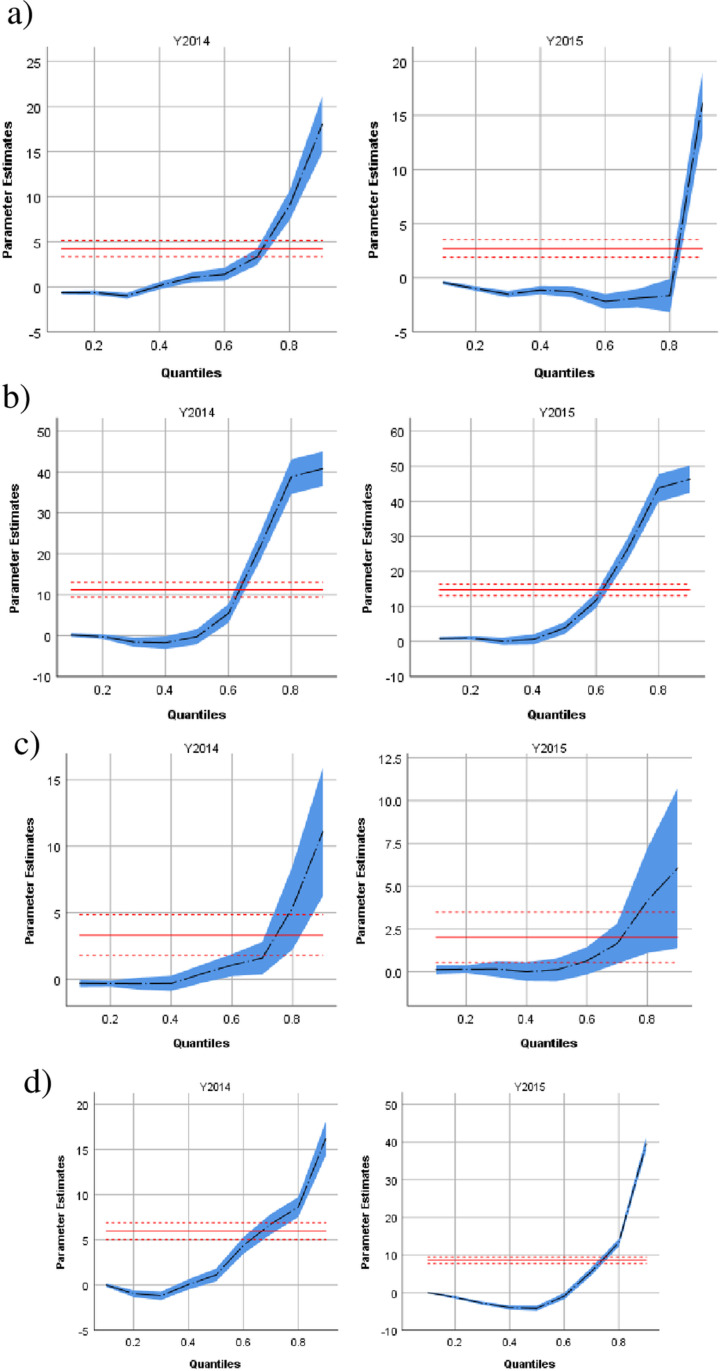
Differences in clinic−specific waiting times across quantiles in the conflict period (2014) and the post−conflict period (2015). The year 2013 serves as the reference year. a) Surgery b) Medicine c) Oncology d) Imaging Parameter estimates for the years are controlled for age, sex, ethnicity and the daily number of visits.

**Fig 3:**
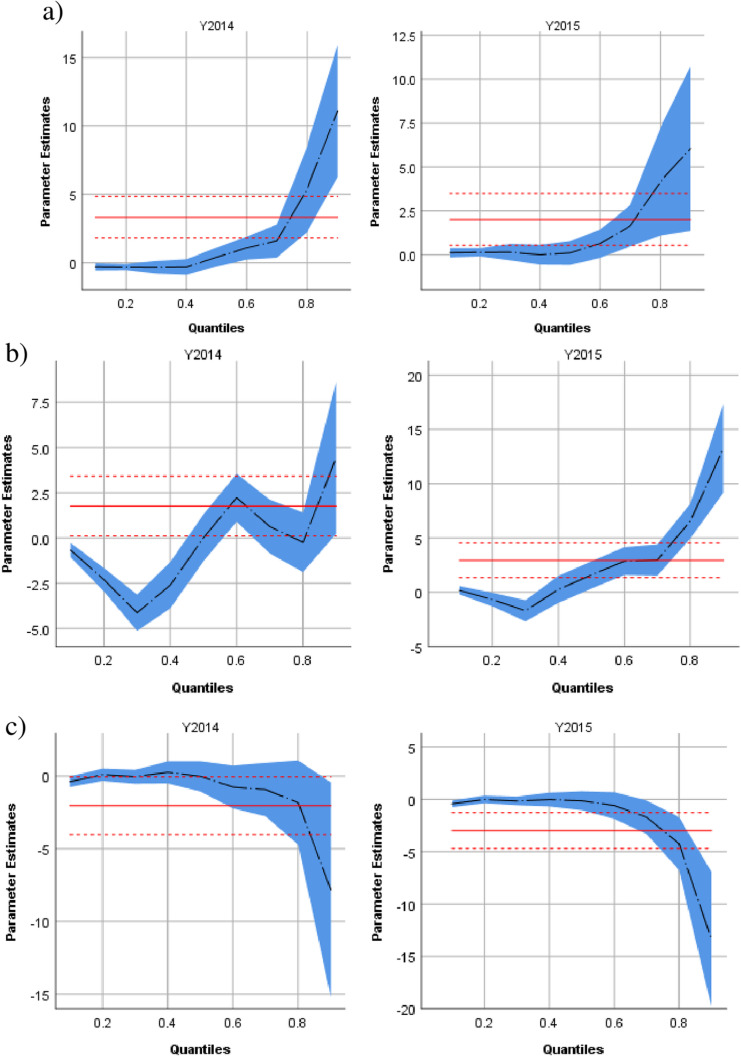
Differences in clinic−specific waiting times across quantiles in the conflict period (2014) and the post−conflict period (2015). The year 2013 serves as the reference year. a)Pediatrics b) Cardiology c) Psychiatry Parameter estimates for the years are controlled for age, sex, ethnicity and the daily number of visits.

When exploring different quantiles of the waiting time distribution (Fig 2 and Fig 3), during PE waiting times in quantiles above the median (50^th^ to 90^th^ quantiles and beyond) consistently increased with a longer delay in the upper quantiles several types of clinics including surgery (up to +17 days for the 90^th^ quantile), medicine (up to +40 days for the same quantile), oncology (up to +12 days) imaging (up to +15 days) and pediatrics (up to +12 days). In contrast, there was no consistent trend in adjusted waiting time in various quantiles for cardiology, while waiting times for psychiatry consistently decreased across quantiles by up to 7 days for the 90^th^ quantile.

The characteristics of outpatient visits during the military operation and during the comparison periods are presented in Table 1. Outpatient visits during the military operation were similar to those occurring during the comparison periods in terms of patient age, sex and ethnicity, although due to the large sample sizes, these differences reached statistical significance.

## Discussion

The main findings of the current study were a decrease in the throughputs of outpatient clinics of about 10% during a regional military conflict. The unadjusted median waiting time was 19 days during PE, vs. 20 days in the pre−conflict period and 18 days in the post−conflict period. There was variance in the behavior of certain clinics, so that the unadjusted and adjusted waiting times for some types of clinics increased, while for other types it was decreased or did not change. The increase in waiting times was more pronounced in certain clinics (e.g., surgery, imaging) than in other (with a decrease in waiting times for pediatrics and no consistent change in other clinics such as cardiology, medicine and psychiatry).

In an extensive search of the literature, we failed to find similar publications dealing with the impact of war on outpatient activity, apart from two papers [20,21] In Ethiopia [20], during the war that broke in November 2020, a sharp 52% decrease in outpatient activity took place. However, there are notable differences between Israel and Ethiopia which make it difficult to compare the two counties. During the 2^nd^ Lebanon War (July to August 2006), a 5% decrease in outpatient activity was noted in Ziv Hospital in Northern Israel [21]. In a previous paper published by our group [13], a decrease of 13% was also noted for emergency department visits. Waiting times increased on average by 0.25 hours even for these emergency cases.

Findings from the study indicating a decline in outpatient activity and a rise in waiting times for particular clinic types can be attributed to a confluence of factors. Concerning demand, patients exhibited reluctance to access the hospital during periods of rocket fire targeting Southern Israel, given that they had only 60 seconds to seek shelter upon hearing sirens, which could potentially occur while *en route* to the hospital. Transportation was also challenged by lockdowns and reduction in the availability of public transportation. Second, many patients were anxious about the war situation and were reluctant to take care of routine medical issues at this time. Challenges related to the supply side were also prevalent. As far as medical manpower is concerned, many physicians were on military service and were therefore absent from the hospital. In addition, of the physicians who were present in the hospital, many were often required to deal with both military and civilian casualties coming to the Emergency Department, especially in surgical specialties. Therefore, their availability for outpatient routine activity was limited. In addition, most buildings used for outpatient visits were unprotected and could not be used for elective activity during times of heavy rockets firing, in accordance with the Homefront Command of the Israeli Defense Force.

It is important to highlight that the escalation in waiting times was not consistent across all clinics. Certain clinics, typically those offering elective procedures such as surgery and imaging, experienced a more significant increase in waiting times, whereas others, notably pediatrics, observed a decrease. This variation can be elucidated by the heightened priority parents place on children’s health, coupled with a reduction in referrals, resulting in enhanced availability for pediatric services. We observed that missed pediatric appointments could have long−term developmental consequences, as children may have lost critical windows for developmental intervention. Studies in conflict zones have shown that disruptions in child health services often lead to adverse outcomes, particularly in developmental care [3,22]. Of note, this study utilized quantile regression to examine various quantiles of the distribution of waiting times. Thus, effects which come into effect only for patients with excessive waiting times (e.g., in the 90^th^ percentile) can be analyzed utilizing a multivariable model, while controlling for confounders. In comparison, linear regression would only model the mean waiting times and therefore miss these outliers completely (see Figs 2–3).

While our study focuses primarily on the impact of the military conflict (PE) on outpatient visit volumes and waiting times, the post−conflict period offers significant insight into the resilience and recovery of the healthcare system. The re−establishment of routine outpatient services in the post−conflict period is critical, as healthcare systems must not only return to pre−conflict functioning but also manage any backlog caused by deferred care during the conflict.

In our data, we observed that outpatient visits during the post−conflict period (2015) rebounded to levels similar to the pre−conflict period, indicating a relatively swift recovery in terms of visit volumes. However, it is important to consider that a full recovery of the healthcare system extends beyond simply matching prior activity levels; it includes addressing increased demands for care resulting from delayed or interrupted treatments, as well as the psychological and logistical challenges faced by both patients and healthcare providers during this period. Additionally, certain specialties such as psychiatry, pediatrics, and surgery may require more targeted efforts in post−conflict recovery due to their higher levels of reduction in patient visits during PE. A deeper analysis of the recovery strategies employed in these disciplines could provide valuable lessons for future conflicts or crises. For example, outpatient services may need to strategize around prioritizing the most urgent cases and expanding capacity temporarily to manage both the immediate post−conflict needs and long−term care demands.

The present study has several strengths. These include the large sample size, allowing for robust statistical analysis; real−life data allowing to test the effect of a “natural experiment”, reliable data coming from the database of SUMC, and the ability to drill down into different types of clinics. However, the study also has some limitations. The study represents the experience of a single center. The external validity of our findings must still be examined using data from other hospitals placed in a similar situation. Similar to our paper on emergency department visits, but even more strongly so, referral bias may have been an issue as patients may have preferred to be treated elsewhere. This possibility was probably tempered by the awareness that prolonged travel during ongoing bombardment incurs additional risk [13]. Information bias might have occurred, as the classification of clinics into groups of similar characteristics may have been somewhat arbitrary and prone to non−differential misclassification.

## Conclusion

The present study offers distinctive insights into waiting times and elective activity within outpatient clinics for patients attending a hospital that serves as a crucial referral center for urgent care amidst a military conflict, all while addressing the routine healthcare needs of its local population. Our findings reveal a decline in activity alongside an increase in waiting times for some types of outpatient visits. Stakeholders should take into account the impact of ongoing military conflicts on non−urgent care and strategize for the restoration of outpatient services as the conflict diminishes.
